# Genetic etiology of adult intellectual disability (ID) of unknown cause in Qatar: a retrospective study

**DOI:** 10.5339/qmj.2022.26

**Published:** 2022-06-14

**Authors:** Hesham Rustom, Yassin Hassan Eltorki, Mohamed Adil Shah Khoodoruth, Oraib Abdallah, Noriya Al-Khuzaei, Noriya Iqbal, Noriya Alabdulla

**Affiliations:** ^1^Psychiatry Hospital, Hamad Medical Corporation, Qatar E-mail: hrustom1@hamad.qa; ^2^College of Medicine, Qatar University, Doha, Qatar

**Keywords:** Intellectual disability, syndrome, Chromosome Disorders, Genetic testing, genetic counseling

## Abstract

Background: Intellectual disability (ID) is a common condition that consists of a heterogeneous group of clinical conditions with different etiologies, including genetic conditions. Identifying those with a genetic cause results in better clinical management.

Aim: To identify the genetic etiology of ID in adult patients with unknown etiology presenting to a specialist learning disability service in Qatar.

Methods: Retrospective review of chart notes of patients referred for ID service from January 1, 2015 to January 1, 2020.

Results: Of the 228 patients, 82 had a known cause of ID and did not require genetic testing, 22 had an unknown cause and underwent genetic testing, and 124 had an unknown cause and did not undergo genetic testing. Of the 82 patients with a known cause of ID, about one-half had an autistic spectrum disorder (ASD) and 18 patients had a genetic disorder. Of the 22 patients who underwent genetic testing, 2 were positive for the Fragile-X mental retardation 1 gene, 3 underwent chromosomal microarray, and 7 underwent whole-exome sequencing. Seven abnormal genes were identified.

Conclusions: Identifying the underlying genetic etiology of patients with ID has major implications for diagnostic and therapeutic approaches. Additionally, it guides a prediction of the natural history of the disease and makes it possible to test at-risk family members.

## Background:

Intellectual disability (ID) is defined as “a significantly reduced ability to understand new or complex information and to learn new skills (impaired intelligence), resulting in a reduced ability to cope independently (impaired social functioning)”. ID begins before adulthood and has a lasting effect on development.^1^ The global estimate of the prevalence of ID is 1%, with a higher prevalence in low- and middle-income countries, including children and adolescents.^2^

The core features of ID disorders are significant limitations in intellectual functioning and adaptive behavioral functioning with onset during the developmental period. ID disorders are further characterized as mild, moderate, severe, or profound levels of severity.^3^

The etiologies of ID include genetic (e.g., Fragile-X syndrome), nutritional (e.g., iodine deficiency), infectious (e.g., intrauterine rubella), metabolic (e.g., phenylketonuria), and neurotoxic conditions (e.g., fetal alcohol syndrome).^4^ The causes of some IDs are well understood. However, the underlying causes of many other IDs (e.g., autism spectrum disorder [ASD]) are often unclear and may vary substantially among individuals.^5^

Genetic testing has been increasingly used in the diagnostic workup of suspected ID. Genetic etiologies can be established by chromosomal microarray (CMA) to detect the copy number variants (CNVs) and regions of homozygosity, by whole-exome sequencing (WES) to detect coding region sequence level variants, CNVs, and the Fragile-X mental retardation 1 gene (FMR1) CCG repeat. The diagnostic yield of clinical WES is 25% in patients with neurodevelopmental disorders and those with ID.^4^ The cumulative diagnostic yield for CMA, WES, and FMR1 is > 50%.^6,7^

Hochstenbach et al. 2011 reviewed the contributions and limitations of genome-wide array-based identification of CNVs for diagnosing patients with mental retardation (ID). A causative genomic gain or loss was detected in 14%–18% of unselected mental retardation referral cases. The expected diagnostic yield for patients with autism is 5%–10% in non-syndromic and 10%–20% in syndromic patients. Exome sequencing in patients with mental retardation or autism reveals *de novo* mutations in protein-coding genes in 60% and 20% of cases, respectively.^8^

Identifying the genetic etiology of ID has several practical advantages, including better clinical management by addressing known comorbidities,^9^ patients and caregivers receive genetic counseling, and patients are in a better position to make informed decisions about their lives.

Unfortunately, the genetic etiology of ID is not always identified in practice. A study in Scotland reported that approximately 14% of the study population who has ID due to a genetic abnormality were identified.^9,10^ Data on the prevalence of ID and the genetic causes are quite scarce in Middle Eastern countries, where the consanguinity rate is very high.^11^

Hamad Medical Corporation (HMC) is the largest governmental healthcare organization in Qatar and the Gulf region.^12^ The learning disability service is based in the psychiatry department and the first clinic was established to offer specialist assessments and treatment for adults with ID in February 2018. By February 2020, the scope of service was planned and adults with mental health issues or challenging behaviors represented the main population. The learning disability community team caseload was 383 cases by 1 August 2021 (247 males and 130 females). The patients were managed using standardized care-planning models in outpatient, community outreach, and hospital consultation liaison settings. The service also offers training and education to other staff and caregivers of ID patients. Referral to the genetic clinic at the HMC is a well-established care pathway between the specialties.^12,13^

## Aim of the study

This study aimed to reflect the current practices at the specialist learning disability service of Qatar for patients with adult ID of unknown cause. Moreover, the study explored the demographics, severity of ID, comorbid medical and psychiatric illnesses, and medications prescribed to patients who underwent genetic testing during the study period. The study was designed to increase awareness of the importance of genetic testing for adults with ID in Qatar.

## Methods

### Study design, setting, and population

The study design was a retrospective chart review of adults with ID who were under care by the ID specialty clinic at HMC in Qatar. The study period was from 1 January 2015 to 1 January 2020. Patients were included if they were 18–65-years-old and presented to the outpatient department of Mental Health Services at the HMC, Qatar. Patients with neurodevelopmental disorders, such as ASD and attention deficit hyperactivity disorder (ADHD) without comorbid ID were excluded.

### Data analysis and interpretation

The data were analyzed with SPSS statistical software (version 24.0; IBM Corp., Armonk, NY, USA). Frequencies and percentages and means ± standard deviations were calculated, as appropriate. A narrative description of the genetic findings was summarized and recorded.

### Ethics approval

The study received ethics approval from the HMC Institutional Review Board (IRB application number: MRC-01-20-02). Individual patient consent was not deemed necessary by the IRB.

## Results

### Causes of ID

A total of 228 patient profiles were reviewed. Eighty-two (36%) patients had a known ID etiology and did not require genetic testing. Twenty-two (10%) patients had an unknown ID etiology and underwent clinical genetic testing. However, 124 (54%) patients with an unknown etiology did not have any documented genetic testing.

### Demographics and clinical characteristics of the patients who underwent genetic testing

Of the 22 patients who underwent genetic testing, 63% were male, 50% were 18–30-years-of-age, 54.4% were Qatari, 27.3% were Arabic non-Qatari, and 18.2% were from the Asian subcontinent (Table 1). Ten (45.5%) patients had moderate ID and four (18.2%) had severe ID. Eight patients had comorbid developmental disorders (ADHD in 2 and ASD in 6). About 77% of the cohort had psychiatric illnesses, and challenging behavior was the most common (n=10). About 27% of patients had epilepsy and 22.7% had other physical disorders, including hypothyroidism, diabetes mellitus, asthma, or von Willebrand disease. Nine had a positive family history of ID either in a sibling (n=7) or an uncle (n=2). About 60% of the cohort had dysmorphic features.

### Patients with an unknown ID etiology who underwent genetic testing

Of the 22 patients with an unknown ID etiology who underwent genetic testing, 3 had normal results. The following genes were identified in 6 patients: cytogenetic band 6q21, PTRHD1, 22q11.23 deletion, Cohen syndrome, CTU2 gene, GABRA1 gene, and tuberous sclerosis. Table 2illustrates the identified genes. Other results were inconclusive or not available in the clinical records.

The 22 patients were offered three tests, including CMA, WES, and FMR1 CCG repeat testing in addition to various tests to screen for any endocrinological or metabolic causes. Three patients had positive results on the CMA, 7 for WES, and 2 on FMR1 CCG repeat testing. Table 3 illustrates the findings of each genetic test.

### Medication management of the patients who underwent **genetic testing**

Of the 22 cases that underwent genetic testing, 17 were managed with medications (**Table 4**) The most prescribed medications were antipsychotics (n=15), followed by antidepressants (n=3), mood stabilizers (n=3), and ADHD medication (atomoxetine) (n=1). Second-generation antipsychotics were prescribed more often than first-generation antipsychotics. Selective serotonin reuptake inhibitors were the most commonly prescribed antidepressant.

### Patients with a known ID etiology

Of the 82 patients with a known cause of ID, half (n=41) had ASD. Genetic syndromes (i.e., Down's syndrome, Ehler-Danlos syndrome, DiGeorge syndrome, Lennox–Gaustau syndrome, Fragile-X syndrome, and Tourette's syndrome) were identified in 18 patients. Cerebral palsy was diagnosed in 9 patients. Brain injury, brain damage, or congenital brain malformations were diagnosed in 5 patients. ADD was diagnosed in 4 patients, homocystinuria in 3, and tuberous sclerosis and neonatal meningitis in 1 patient each.

## Discussion

This is the first study in Qatar to examine patients with ID and genetic syndromes. No specific epidemiological data are available; however the prevalence of ID in Qatar is 2%–3% of the general population. Qatar's 2020 population was estimated to be 2,881,053 and Qatari citizens represent 11% of the total population. Approximately 6,000–9,000 Qatari citizens have been diagnosed with IDs and 2,000 cases/people from other nationalities, most were single workers. Therefore, a national ID register for ID patients needs to be established in Qatar.^13^

The majority of the patients were male (14/63.6%), which was attributed to the fact that 72.1% of Qatar's population consists of males.^14^ Half of the studied subjects were young (18–30-years-old) and received a diagnosis of a moderate learning disability (45.5%). However almost half of the patients in the UK exhibit mild disability.^15^ Almost half of the study patients were Qatari nationals. This agrees with the Qatar statistics where 57% of the people with learning disabilities in Qatar are nationals.^16^ Antipsychotic medications were the most commonly prescribed, which requires addressing in the light of well-reputed established guidelines. Approximately 77% of patients were diagnosed with mental illness and were receiving antipsychotics, which is high, compared to patients in Wales where an audit of adults revealed that 20%–29% of patients with ID were prescribed antipsychotics.^17^

Psychiatric diagnoses were present in about three-fourths of patients who underwent genetic testing during the study period, highlighting the importance of managing psychiatric comorbidities in this group of patients through collaborative work among various professionals and resource allocations. Challenging behavior is a common compelling psychiatric diagnosis, as it is estimated to affect 5%–15% of the general LD population. Furthermore, the risk is as high as 40% in young, hospitalized patients with LD. Therefore, the NICE guidelines emphasize the significance of behavioral, psychological, and environmental support plans before advising a psychotropic intervention.^18^ Similar to our current findings, the epilepsy society has reported that 1 in 3 patients with LD suffers from epilepsy.^19^

A growing body of literature underscores the importance of determining the genetic etiology of ID, as it offers a sense of empowerment and clarity to patients, determines the clinical course and prognosis, helps identify and manage medical comorbidities, and allows patients to receive specific counseling and plan their future.^7^ About 40% of our ID sample had a family history of ID, which further emphasizes the importance of genetic testing and the need for families to receive genetic counseling. However, in our study of adult patients, the etiology of ID was not identified in more than half of the patients. This may be due to the later introduction of the genetic screening program for newborn babies,^20^ limited local availability of genetic testing, differences in practices of frontline ID clinicians and the Clinical and Metabolic Genetics department, lack of collaborative working protocols, and issues around patient capacity and consent. Studies indicate that 6% of adult patients accepting learning disability services in the study area have a known genetic abnormality. Most of the patients in their sample who have had genetic testing were tested as children, or before the recent advances in genetic testing using the CGH array.^9^

The effect of family history on learning disabilities is not negligible. Snowling et al. 2003 reported a continuous, high risk of developing a learning disability during childhood and followed up the results (Snowling et al. 2007) during adulthood. About 66% of high-risk children (children born to families with a history of learning disability) have a diagnosis of a learning disability compared to 13% of the control group.^21,22^

Further studies at the Clinical Genetics Department at the Western General Hospital in Edinburgh (UK) indicate that approximately 20% of patients with an ID of unknown cause have pathogenic genetic abnormalities.^23-26^ Michelson et al. (2011) systematically reviewed the evidence concerning the diagnostic yield of genetic and metabolic evaluations of children with general developmental delay and ID. As results, microarray testing was diagnostic in 7.8% of cases, G-banded karyotyping was abnormal in at least 4%, and fluorescence *in situ* hybridization was positive in 3.5%. Testing for X-linked ID genes has a yield of up to 42% in males with an appropriate family history.^27^ Sagoo et al. (2009) performed a systematic review that evaluated array-based comparative genomic hybridization used in patients with ID and congenital anomalies. The findings were that the overall diagnostic yield of causal abnormalities was 10%. The overall number needed to test to identify an extra causal abnormality was 10.^28^

Most patients with suspected genetic inheritance are referred for investigation. The benefit from those referrals is quite evident but some families are hesitant to cooperate due to the social stigma. Other cultural factors influence the family's decision to participate in this process. Stigma is related to mental illness and is one of the main factors preventing people from seeking help in Qatar.^29^

There is a need to raise awareness among clinicians and patients about the importance of undertaking genetic testing and establishing clear local pathways for this specialist group. ID teams need to consider offering genetic testing to ID patients with unknown etiology.

However, universal genetic testing is not being recommended due to the high cost, lack of universal access to testing, the ethical issues of dealing with an unrelated diagnosis, and the stress and uncertainty among close relatives.

The results show that 50% of patients with a known cause of ID have Asperger (highly functioning autism), autism, or some form of ASD. It will be interesting if future research includes these patients for genetic testing.

One of the limitations of this study was that we could not obtain the data of some of the ID patients referred for genetic testing in Qatar. This could be due to a lack of clear record-keeping or a delay in some of the genetic results in the electronic system. In addition, some of these tests were performed in the USA, which delayed receiving the results. Some missing results, such as 50% (11/22) of the WES results, could not be found. The WES test was not performed in Qatar but was sent to the USA and the results may not have been documented in electronic records, which could be a contributing factor.

## Conclusion

Identifying the underlying genetic etiology of patients with ID can have major implications for diagnostic and therapeutic approaches, can guide the prediction of the natural history of the disease, and make it possible to test at-risk family members.^30-35^

To ensure high-quality care, there is a need for introducing collaborative practice pathways that ensure that genetic testing is routinely offered to patients with ID who have dysmorphic features or who have an unknown underlying etiology.^36^

We also suggest the development of an ID patient registry for genetic testing to assist with future testing in light of advancements in the field and to assist with future research.

The adult learning disability services in Qatar should consider genetic testing in all patients with an ID of unknown etiology.

Therefore, it has been agreed among professionals working in the learning disability service at HMC that those patients would be referred for genetic testing and undergo the WES. If an abnormality is identified, the patient and family will receive counseling from specialists at the HMC genetic and metabolic clinic and discuss the implications.

### Data availability

The data that support the findings of this study are available from the corresponding author upon reasonable request and pending additional ethical approval.

### Author contributions

All authors made substantial contributions to the conception and design of the study, data collection, analysis, drafting, revising of the article, and approval of the final version for submission.

### Funding

Hamad Medical Corporation agreed to contribute toward the publication fee.

### Declaration of competing interest

The authors declare no competing interests.

## Figures and Tables

**Table 1:** Demographics and clinical characteristics (N=22) of the patients who underwent genetic testing

Parameters	n (%)
**Gender**	
Female	8 (36.4)
Male	14 (63.6)
**Age (Years)**	
18-30	11 (50)
31-40	7 (32)
41-50	4 (18)
Ethnicity	
Arabic non-Qatari	6 (27.3)
Arabic Qatari	12 (54.5)
Asian subcontinent	4 (18.2)
**Severity of ID**	
Mild	8 (36.4)
Moderate	10 (45.5)
Severe	4 (18.2)
**Developmental disorder**	
ADHD	2 (9.1)
ASD	6 (27.3)
None	14 (63.6)
**Psychiatric diagnosis**	
No	5 (22.7)
Yes	17(77.3)
*Challenging behavior; 10 (58.8)*	
*Anxiety Disorder; 2 (11.8)*	
*BAD; 1 (5.9)*	
*Post-traumatic stress disorder; 1 (5.9)*	
*Schizophrenia and schizoaffective disorder 3 (17.7)*	
**Physical disorder**	
No	11 (50)
Epilepsy	6 (27.3)
Others (hypothyroidism, Diabetes Mellitus, asthma or/and von Willebrand disease)	5 (22.7)
**Family History of intellectual disability**	
No	13 (59.1)
Yes	9 (40.9)
**Dysmorphic features**	
No	9 (40.9)
Yes	13 (59.1)
**Receiving Psychotropic medications**	
No	5(22.7)
Yes	17(77.3)
**Prescribed medications**	
Antipsychotics *[Aripiprazole (2), Chlorpromazine (1), Risperidone (5), Olanzapine (5), Haloperidol (1), Quetiapine (1)]*	15 (68.2)
Antidepressants *[(Fluoxetine (2) and escitalopram (1)]*	3(13.6)
Mood stabilizers *[Lithium (2) and Carbamazepine (1)]*	3(13.6)
AntiAttention Deficit Hyperactivity disorder (*Atomoxetine*)	1 (4.5)

Legend: n= number of respondents

**Table 2:** Identified genes

1	n
cytogenetic band 6q21	1
PTRHD1	1
deletion of 22q11.23	1
Cohen syndrome	1
CTU2 gene	1
GABRA1 gene	1
Tuberous sclerosis	1

**Table 3:** CMA, WES, and FMR1 CCG repeat analysis results

Results	n (%)		
	Negative	Not found	Positive
**CMA**	13(59.1)	6(27.3)	3(13.6)
**WES**	4(18.2)	11(50)	7(31.8)
**FMR1 CCG Repeat (Fragile-X)**	15 (68.2)	5(22.73)	2(9.09)

Legend: CMA chromosomal microarray; WES = whole-exome sequencing, FMR1= Fragile-X mental retardation 1 gene
